# Distributed Raman Spectrum Data Augmentation System Using Federated Learning with Deep Generative Models

**DOI:** 10.3390/s22249900

**Published:** 2022-12-16

**Authors:** Yaeran Kim, Woonghee Lee

**Affiliations:** 1 Division of Computer Engineering, Hansung University, Seoul 02876, Republic of Korea; 2 Smart CBRNe Sensor Laboratory (SCSL), Seoul 02841, Republic of Korea; 3 Department of Applied Artificial Intelligence, Hansung University, Seoul 02876, Republic of Korea

**Keywords:** Raman spectrum, data augmentation, distributed system, federated learning, deep generative model

## Abstract

Chemical agents are one of the major threats to soldiers in modern warfare, so it is so important to detect chemical agents rapidly and accurately on battlefields. Raman spectroscopy-based detectors are widely used but have many limitations. The Raman spectrum changes unpredictably due to various environmental factors, and it is hard for detectors to make appropriate judgments about new chemical substances without prior information. Thus, the existing detectors with inflexible techniques based on determined rules cannot deal with such problems flexibly and reactively. Artificial intelligence (AI)-based detection techniques can be good alternatives to the existing techniques for chemical agent detection. To build AI-based detection systems, sufficient amounts of data for training are required, but it is not easy to produce and handle fatal chemical agents, which causes difficulty in securing data in advance. To overcome the limitations, in this paper, we propose the distributed Raman spectrum data augmentation system that leverages federated learning (FL) with deep generative models, such as generative adversarial network (GAN) and autoencoder. Furthermore, the proposed system utilizes various additional techniques in combination to generate a large number of Raman spectrum data with reality along with diversity. We implemented the proposed system and conducted diverse experiments to evaluate the system. The evaluation results validated that the proposed system can train the models more quickly through cooperation among decentralized troops without exchanging raw data and generate realistic Raman spectrum data well. Moreover, we confirmed that the classification model on the proposed system performed learning much faster and outperformed the existing systems.

## 1. Introduction

In today’s battlefields, chemical agents are very dangerous weapons that threaten soldiers. Chemical agents have been developed in the past and continue to emerge as new substances. Thus, it is important to detect chemical agents rapidly and accurately on battlefields. Raman spectroscopy is a widely used method for detection. Raman spectrum is obtained from Raman scattering, which is a kind of inelastic scattering phenomenon [1]. It is possible to obtain information on a chemical by analyzing the scattered light emitted when a single color light is projected on the chemical sample. In Raman spectroscopy, the degree of shift compared to Rayleigh scattering is expressed as Raman shift, and the Raman spectrum has peaks in various forms depending on the amounts of scattered photons. Such peaks’ distinguishing forms can be used to identify the structural components of the chemical sample [2]. In addition, Raman spectrum data can be measured non-destructively and easily, regardless of the condition of the sample.

For the reasons mentioned above, Raman spectroscopy-based detectors are widely used these days. However, such chemical agent detection has the following limitations. Raman spectrum sensitively reacts to various environmental factors, such as temperature and moisture. For example, noise can be added to the spectrum depending on the roughness of the material’s surface [3]. Further, there can be variations in the spectrum if the distance between the material and the light source changes. Thus, a false alarm may occur in unpredictable field environments [4]. Moreover, chemical agents are fatal, so it is not easy to produce and handle them, which causes difficulty in securing data about chemical agents in advance. It is hard for the existing detectors to make appropriate judgments without prior information. Due to the aforementioned challenges, the existing detectors with determined rule-based techniques cannot deal with such problems flexibly and reactively.

Artificial intelligence (AI)-based detection techniques can be good alternatives to the existing rule-based techniques for chemical agent detection. In order to train learning models for detection well, it is required to gather large amounts of Raman spectrum data that are realistic while having some differences, not the same data. However, it is not easy to obtain a large number of different Raman spectrum data of fatal chemical agents. To overcome such limitations, in this paper, we devise the distributed Raman spectrum data augmentation system, which leverages federated learning (FL) with deep generative models, such as generative adversarial network (GAN) [5] and autoencoder. The proposed system utilizes various techniques in combination to generate a large number of Raman spectrum data with reality along with diversity. First, a one-dimensional (1-D) Signal GAN is the cardinal technique in the system. The GAN is used to generate Raman spectrum data in large quantities from a small number of real data. However, there is the limitation that the generated data are very similar to each other due to the lack of real data used to train the GAN. Thus, the system utilizes random transformation-based data augmentation (DA) [6] to improve the diversity of GAN. In addition to this, to improve the reality of the data, the system uses a denoising autoencoder (DAE) [7] to remove the exaggerated noise. Moreover, the zero padding (ZP) technique is used to eliminate the artifacts due to the discontinuity feature of the Raman spectrum. Furthermore, we leverage FL to enable the above operations to be performed in a distributed manner, which improves the proposed system’s practical feasibility on real battlefields. We implemented the proposed system and conducted various experiments to validate our system. The evaluation results proved that the proposed system can train the models more quickly through cooperation among decentralized troops without exchanging raw data and generate realistic Raman spectrum data well. Moreover, we confirmed that the classification model of the proposed system outperformed the existing models, which validated the effectiveness of the proposed system. As far as we know, this research is the first to exploit FL with GAN and DAE using the Raman spectrum of chemical agents.

This paper is organized as follows. In Section 2, we introduce the studies related to our research. In Section 3, we describe the application concept and the overall operations of the proposed system and then give detailed explanations about the system design and components. After that, in Section 4, we explain the implementation of the techniques used in the system. In Section 5, we describe the experiments and evaluation results. Finally, Section 6 concludes this paper by explaining remarks and future directions.

## 2. Related Work

This section describes various studies related to our proposed system. We first introduce the studies that have utilized Raman spectroscopy to analyze chemical agents. Then, we explain some studies focusing on applying deep learning (DL) methods to Raman spectrum data. After that, we give descriptions of FL used for distributed systems and military applications. Finally, we explain the models and techniques utilized in the proposed system, and then describe our research’s novelties and advantages in comparison with the relevant studies.

Raman spectroscopy has been applied to the detection of hazardous chemicals for military operations [8], and there were some studies related to this. Yu et al. proposed a baseline correction algorithm that removes the baseline for chemical agent detection while minimizing the distortion of the Raman scattering spectrum [9]. Choi et al. measured the Raman spectra of 18 chemical warfare agents by using 248-nm UV Raman spectroscopy and analyzed the spectral characteristics of each agent [10]. Hu et al. analyzed the spatial heterodyne Raman spectrometer and showed that the equipment has the ability to detect simulants of chemical warfare agents [11].

Supported by advances in DL techniques, various studies leveraged DL methods to improve Raman spectroscopy and analyze Raman spectra. Fan et al. proposed a DL-based approach to identify components in mixtures using Raman spectra, and their scheme showed better sensitivity compared to other machine learning (ML)-based techniques [12]. Weng et al. proposed a DL-based method to recognize drugs in human urine, and the results showed that their neural network model performed better than the common ML methods [13]. Horgan et al. presented a comprehensive framework for higher-throughput molecular imaging via DL-enabled Raman spectroscopy trained on a large dataset of over 1.5 million Raman spectra [14]. Frischia et al. proposed a pipeline for augmenting data using GAN reinforcement [15], and Ma et al. demonstrated a spectral recovery conditional GAN to reduce the data acquisition time [16]. The above studies used DL for applications using Raman spectrum data of non-hazardous substances, not of fatal chemical agents. In [17], an approach utilizing ML was proposed for chemical agent detection, but this approach did not use Raman spectrum data. In addition, the above studies trained their models using a very large number of Raman spectrum data. Specifically, the network model proposed in [15] aimed for data augmentation of the Raman spectrum, but training using thousands of Raman spectra should be required to perform data augmentation. However, in the military field, many tasks frequently require the recognition of rare or never before seen samples [18].

Using FL, devices participating in learning do not need to send raw data to the server, which improves security and privacy and reduces communication resource usage. Thus, FL is a suitable learning method for distributed systems, and many researchers have tried to leverage FL to perform DL in distributed systems. Chen et al. presented an FL-based intrusion detection algorithm to ensure the security of wireless edge networks [19]. Wang et al. proposed an FL-based pedestrian detection scheme that gathers data from multiple vehicles to achieve secure multi-party computation in vehicular scenarios [20]. Sharma et al. proposed a distributed computing defense framework using FL to resolve the challenges of limited training data and avoid a reason-specific model [21]. On battlefields, devices are usually distributed, and communication resources are not abundant. In addition, security is very important, so strong encryption is essential for every data transmission. Therefore, several studies have utilized FL for military uses [18], but there are not many studies yet.

To generate Raman spectrum data properly, the proposed system utilizes various neural network models and techniques. First, the DA is used to secure sufficient amounts of initial data to train the GAN. DA is a universal model-independent data side solution that can help networks overcome small datasets [22,23]. After that, the system utilizes the GAN to generate many, and more diverse data. GANs aim to generate fake data by training a pair of competing networks, the generator and the discriminator. GANs are used in a variety of fields, such as image synthesis, semantic image editing, style transfer, image super-resolution, and classification [24,25]. In addition, the system utilizes the DAE to alleviate the excessive noise included in the data generated by the GAN. DAE is a technique widely used to reduce noise, and it is trained to reconstruct clean results from corrupted inputs by modifying the autoencoder [26,27]. Moreover, our system utilizes ZP, which is a technique that fills the edge of the data with zero values. ZP has been mainly used to make input and output sizes the same by preventing the size of output data from decreasing [28,29]. However, our system utilizes the ZP in a way that is different from the ordinary ways other researchers used ZP. By utilizing the ZP, the proposed system makes the artifacts occur on the padded region, and then the system removes the region, which eliminates the artifacts without decreasing the size of the resulting data.

This paper has novelty and advantages compared to the related studies. Some related studies applied DL to Raman spectrum data, but there are few studies using DL for the data of chemical agents. In addition, many of the existing studies used large amounts of data to perform learning, and one of them used over 1.5 million Raman spectrum images [14]. However, it is not easy to generate Raman spectrum data of chemical agents, and it also takes a considerable amount of time. Therefore, it is challenging to generate sufficient amounts of data for training. Furthermore, we leverage FL to enable the proposed system to operate in distributed environments, which improves the system’s practical feasibility on real battlefields.

## 3. System Design

In this section, we first describe the concept of the distributed Raman spectrum data augmentation system and then we explain the overall design and the operations of the proposed system. After that, we give detailed explanations about the techniques used for the system.

### 3.1. System Concept

**Figure 1:**
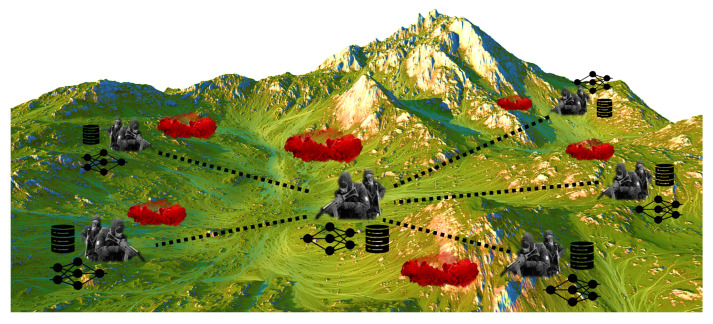
The application concept of the proposed system.

Chemical attacks are a significant threat in modern warfare. Specifically, it is important to respond quickly to the simultaneous chemical attacks in different regions, as shown in Figure 1. If there is a model that has already been trained sufficiently in advance, immediate responses to the chemical attacks are possible, but if not, training a model to identify the chemical agent should be performed quickly. However, due to the danger of chemical agents, it is not easy to generate Raman spectrum data of chemical agents, and it also takes a considerable amount of time. Therefore, it is challenging to generate the data required for training. Motivated by this, we devise a system that generates sufficient amounts of Raman spectrum data based on a small number of collected real data, which properly reflects diverse changes while maintaining the characteristics of the real data. Figure 1 shows the proposed system’s application concept. The proposed system utilizes not only deep generative models, such as GAN and DAE, but also additional techniques, such as the random transformation-based DA and ZP, to generate Raman spectrum data properly. Furthermore, the system leverages FL to enable cooperation among decentralized troops and faster learning. Using FL, it is possible to build a global model more quickly without exchanging raw data between the troops, as shown in the figure.

### 3.2. Overall Design

In this subsection, we describe the overall procedure of the proposed system and give brief descriptions of each process. The proposed system should be able to mass-produce data that include variations while having a certain level of similarity with the existing real data. To meet such requirements, the system includes two training processes to generate data that satisfy the conditions.

**Figure 2:**
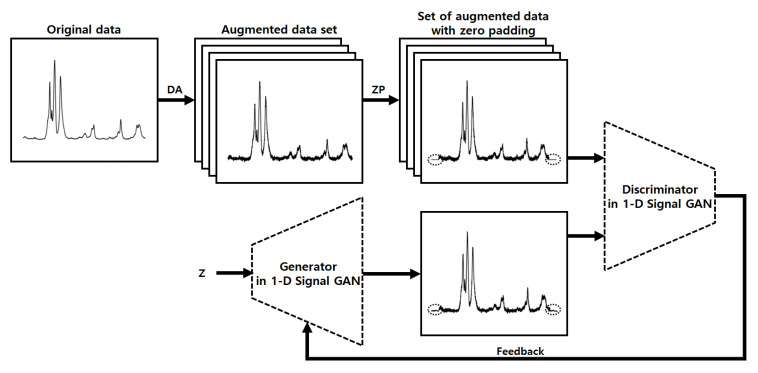
The first training process in the proposed system.

Figure 2 shows the first training process in the proposed system. The DA based on random transformation is the first step in the first training process. Noise and shifting can be found in Raman spectrum data being influenced by the surrounding environments. Thus, the system performs DA to create more diverse data by reflecting the appropriate Gaussian noise and shifting in the real data. After that, the system utilizes a 1-D signal GAN to generate many and more diverse data. General GANs optimized for 2-D image generation are not suitable for 1-D data, such as Raman spectra, so we chose to utilize the 1-D signal GAN derived from the typical GANs in our system. If the GAN is trained using only a small number of real data, the GAN model is overfitted to the training data. The model trained in this way creates only the same data as the original data and cannot generate diverse data affected by various environmental factors. Therefore, using the aforementioned augmented data, the system trains the GAN to create data that maintain the characteristics of the original data but differ to some extent.

**Figure 3:**
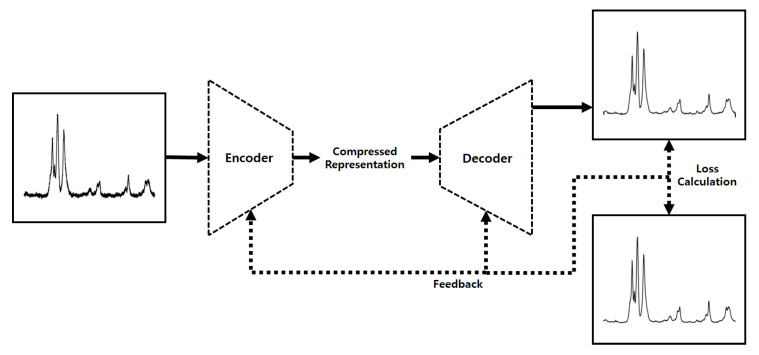
The second training process in the proposed system.

As we explained above, the augmented data are used as the training data to improve the diversity of the GAN, but as a side effect of this, the GAN generates noise-enhanced data. Thus, the system utilizes DAE to alleviate the excessive noise included in the generated data. Similar to the autoencoder, DAE includes an encoder and a decoder, but there are some differences in the learning process. Figure 3 shows the training procedure of DAE, which is the second training process in the proposed system. We will give a detailed explanation of the training for DAE in Section 3.5.

**Figure 4:**
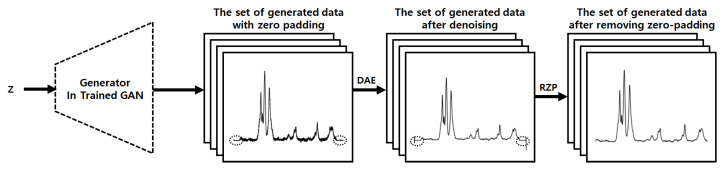
The generation process in the proposed system.

Using the trained GAN and DAE, the system generates various types of data, including reasonable variations. However, the generated data contains unnatural soaring values at both ends due to the discontinuity at both ends of the data used for training. To solve this problem, the system uses data with some padding values added to both ends as the training data, as shown in Figure 2. After data generation and denoising, the system removes the values added in the padding regions to get rid of the aforementioned artifacts and finally completes the process. Figure 4 shows the generation process of the proposed system. We will present the detailed results of each step in Section 5.

Through the above operations, the system is able to obtain a large number of data that properly reflect diverse changes while maintaining the characteristics of the original data.

### 3.3. One-Dimensional Signal Generative Adversarial Network

As explained in Section 3.2, other steps, such as the DA and the ZP, exist before training the GAN, but we first explain the GAN to make it easier to understand the context between the consecutive steps.

GAN is the main technique of the proposed system. We describe the mathematical explanation of the GAN with reference to [5,30,31]. The GAN is composed of two models, a generative model, *G*, and a discriminative model, *D*. The loss function is derived from the binary cross-entropy loss as follows:

(1) $$L\left(\hat{y},y\right)=\left[y\log\hat{y}+\left(1-y\right)\log\left(1-\hat{y}\right)\right].$$

While training the discriminator, the label of data from the original data distribution, $P_{data}\left(x\right)$, is *y* = 1 (i.e., real) and $\hat{y}$ = D(x), and Equation (2) is derived by substituting these into Equation (1).

(2) L(D(x),1)=log(D(x)).

Similarly, when the label is *y* = 0 (i.e., fake data) and $\hat{y}$ = D(G(z)) for data from the generator, we can derive Equation (3).

(3) L(D(G(z)),0)=log(1−D(G(z))).

In order to classify the fake and the real, Equations (2) and (3) should be maximized as follows:

(4) $$L^{D}=\max\left[\log\left(D\left(x\right)\right)+\log\left(1-D\left(G\left(z\right)\right)\right)\right].$$

On the contrary, the generator tries to minimize the loss as follows:

(5) $$L^{G}=\min\left[\log\left(D\left(x\right)\right)+\log\left(1-D\left(G\left(z\right)\right)\right)\right].$$

To consider the entire dataset, we take the expectation of the combined form of Equations (4) and (5) as follows:

(6) $$\min_{G}\;\max_{D}\left[E_{x∼P_{data}\left(x\right)}\left[\log D\left(x\right)\right]+E_{z∼P_{z}\left(z\right)}\left[\log\left(1-D\left(G\left(z\right)\right)\right)\right]\right],$$

where $P_{z}\left(z\right)$ denotes the input noise distribution.

The proposed system utilizes a 1-D signal GAN to generate a large number of Raman spectrum data. In Raman spectrum data, the *x*-axis refers to the Raman shift and the *y*-axis means the intensity of the Raman spectrum obtained at each Raman shift value. Raman spectra are data in 1-D form, so we needed GANs suitable for such forms to augment the data. In general, widely used GANs are 2-D GANs suitable for images, and the 2-D convolution matrices are used for convolution calculations for the image data. If Raman spectra are deemed images, the 2-D convolution can be applied to Raman spectrum data. However, unlike typical images, the Raman spectrum image has almost all areas of the image filled with white, and the line that is the intensity values is only a small fraction of the data. Thus, the 2-D convolution is not suitable for Raman spectra in 1-D data form. In addition, since Raman spectra are not data with temporal flows, such as a stock chart, it is not appropriate to apply recurrent neural network (RNN)-based models, such as long short-term memory (LSTM) and gated recurrent units (GRU). For these reasons, we found that the 1-D signal GAN using Conv1d operations is suitable for the augmentation of Raman spectrum data, so the proposed system utilizes the 1-D signal GAN.

### 3.4. Random Transformation-Based Data Augmentation

The performance of the neural network model can significantly depend on how much data are used for training, and the larger the number of various training data, the better overfitting can be avoided. In other words, overfitting should occur when the GAN is trained using only a small number of real data. Actually, when the 1-D signal GAN was trained using only a small number of data, the trained GAN generated only very uniform data, which limited the diversity of the GAN. Therefore, the proposed system leverages the DA to generate various initial training data with a certain level of change by shifting or adding noise to a small number of real data. DA is a technique to generate larger amounts of new data by changing existing data [32]. The system utilizes the random transformation-based DA, which is suitable for Raman spectrum data because such a technique can be applied to data that have a shape similar to the Raman spectrum. There are various methods in the random transformation-based DA, and among them, the methods applicable to the 1-D signal are jittering, scaling, rotation, permutation, magnitude warping, time warping, cropping, flipping, and window slicing [22]. Among these methods, the proposed system performs DA using jittering and shifting because such methods can mimic the changes that frequently occur in Raman spectrum data due to environmental influences.

Gaussian noise is the most commonly known noise that exists in all frequency bands. This noise is easily seen in nature and also exists in the Raman spectrum data [33], so jittering with Gaussian noise was chosen as one of the DA methods. Shifting is an augmentation method that moves each value of data in a certain direction without modifying the overall form of the data. Thus, it can be utilized to imitate the changes in the peaks’ location of the Raman spectrum due to the influence of the environment when measuring a chemical. The shifting moves values in a vertical or horizontal direction in general, but only the horizontal moving was used in the proposed system because the peaks in the Raman spectrum generally move horizontally.

Using the above methods, the system performs DA using a small number of real data to secure sufficient amounts of initial data for training the GAN.

### 3.5. Denoising Autoencoder

**Figure 5:**
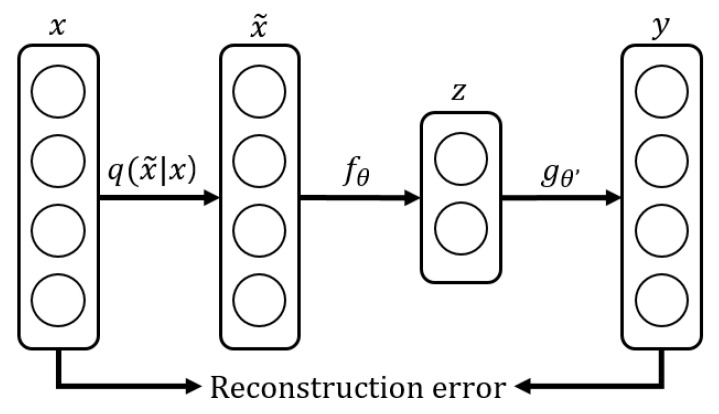
The mathematical operation flow in DAE.

DAE has the same encoder and decoder as the autoencoder, but there are some differences in the learning process, represented in Figure 3. We describe the mathematical explanation of DAE with reference to [34]. Figure 5 briefly shows the overall flow of the mathematical operations in DAE. First, the random noise following the probability distribution, $q(\tilde{x}|x)$, is added to an input vector, *x*. Then, the encoder with the parameter $f_{\theta }$, uses $\tilde{x}$ as an input and outputs a latent vector, *z*. Similarly, the decoder with the parameter $g_{\theta^{\prime }}$, outputs *y* by using *z*, and the difference between *x* and *y* is the reconstruction error. This loss can be minimized by optimizing the parameters of the encoder and the decoder as follows:

(7) $$\underset{\theta ,\theta^{\prime }}{\arg\min}\left[E_{q^{0}\left(X\right)}\left[L\left(X,g_{\theta^{\prime }}\left(f_{\theta }\left(X\right)\right)\right)\right]\right],$$

where $q^{0}\left(X\right)$ denotes the empirical distribution-associated *n* training inputs, and L(x,y) is a negative log-likelihood for *x*, given *y*.

DAE is trained in a way that the decoder outputs data as similar as possible to the original data when the original data with noise are provided to the encoder as input. After DAE is trained sufficiently, DAE is able to remove the noise from the noisy input data [7]. As explained in Section 3.4, the proposed system uses augmented data to improve the diversity of the GAN. However, this process causes the side effect that the GAN generates data with amplified noise. This noise is exaggerated compared to the noise obtained in nature, so the proposed system performs denoising on it by using the trained DAE.

### 3.6. Zero Padding for Removing Artifacts Due to Discontinuity

The system with the trained GAN and DAE generates new data mimicking the real data well. However, due to the discontinuous parts at both ends of the Raman spectrum, the generated data include unintended artifacts. To solve this problem, the proposed system utilizes the ZP technique. Instead of using the augmented data as they are, the system adds zero values of a certain length to both ends of the augmented data and uses the zero-padded augmented data for training. The system with the trained models generates new Raman spectrum data that are longer than the original real data and include the artifacts at both ends. Therefore, the system removes as many values as the length of the previously added padding at both ends of the generated data, as shown in Figure 4. This removal operation not only makes the length of the generated data the same as the original length but also eliminates the artifacts.

### 3.7. Federated Learning for Distributed Raman Spectrum Data Augmentation System

**Figure 6:**
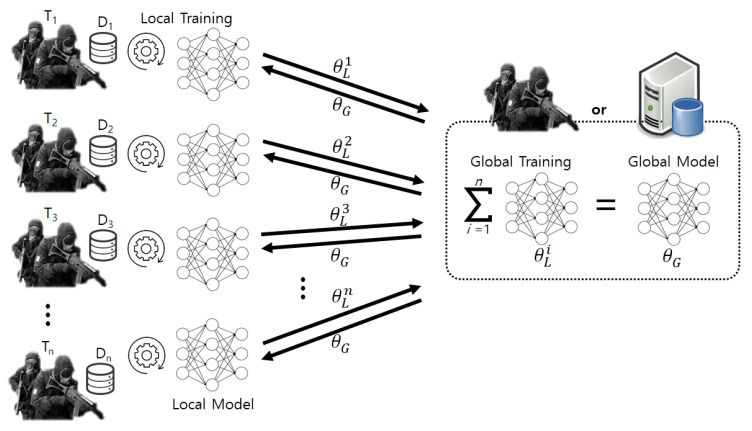
The overall operations of FL in the proposed system.

As explained before, the proposed system leverages FL to enable cooperation among decentralized troops and faster learning. The proposed system conducts FL by utilizing federated averaging [35], which is the most widely used FL algorithm. Figure 6 shows the overall operations of FL in the system. In order to explain the FL operations, we assume that there is one server and *n* troops, $T_{1}$, …, $T_{n}$, which have their own Raman spectrum dataset, $D_{1}$, …, $D_{n}$. In this situation, the FL in the system includes the following major steps. First, the server, headquarters, or one of the troops, sends the initial global model (i.e., the generator and discriminator models of GAN or the encoder and decoder models of DAE) to all of the troops. Then, each troop trains their local models using local Raman spectrum data as follows:

(8) $$\theta_{L}=\theta_{L}-\eta g_{k},$$

where $\theta_{L}$, η, and $g_{k}$ denote the local model, the learning rate, and the gradient, respectively. After that, the troops send their local model’s parameters, $\theta_{L}^{1}$, …, $\theta_{L}^{n}$, back to the server, and the model parameters are aggregated into the global model in the server as follows:

(9) $$\theta_{G}=\sum_{i=1}^{n}\frac{s_{i}}{s}\theta_{L}^{i},$$

where $\theta_{G}$, *s*, and $s_{i}$ denote the global model, the total number of samples, and the number of samples on the *i*th troop, respectively. Then, the global model’s parameters are delivered to the troops again. The above procedures are repeated until the global model is trained sufficiently.

For instance, in the case of GAN, the server aggregates the local generator models’ parameters into the global generator model, $\theta_{g}=\sum_{i=1}^{n}\frac{s_{i}}{s}\theta_{g}^{i}$. Similarly, the server also integrates the local discriminator models’ parameters into the global discriminator model, $\theta_{d}$. After that, the server delivers the global generator and discriminator models back to the troops for the following learning in the next round.

## 4. Implementation

This section gives detailed descriptions of the implementation of the techniques used in the proposed system.

### 4.1. Implementation of Data Augmentation

The DA module was implemented to generate the data according to the selected augmentation method and the number of data. When the original Raman spectrum is provided as input, the DA module performs jittering and shifting on the original to generate a designated number of augmented data. In jittering, the DA module generates Gaussian noises of deviation ranging from 0 to a predetermined value and adds them to the original data. In shifting, the data are moved according to a randomly selected direction and value within a determined scale, and then the empty data fields caused by the movement are filled with zeros.

### 4.2. Implementation of 1-D Signal GAN

We built the GAN by using the PyTorch library [36] with reference to [37]. We utilized Conv1d of PyTorch to build the 1-D signal GAN based on Wasserstein GAN [38]. To be compatible with data of any size, we implemented the input layer width of the discriminator and the output layer width of the generator to be flexibly adjusted depending on the size of the input data. Table 1 and Table 2 show the network structure of the discriminator and the generator in the GAN, respectively. As shown in the tables, the discriminator in the GAN we implemented has five layers, and the input data are converted into the value distinguishing whether the input data are real or fake by going through the layers. The generator also has five layers, but unlike the discriminator, the input noise becomes fake data that are similar to the real data by passing through the layers. The augmented 1-D signal data with zero padding were used as input data to train the generator and discriminator networks. We set the hyperparameters as shown in Table 3 by referring to the values widely used for GANs [37,39]. The size of the dataset and the number of epochs vary depending on the experiments and evaluations. Thus, Table 3 does not include these values, but instead, the descriptions in Section 5 include such information. After training, the system utilizes only the generator model to create Raman spectrum data.

### 4.3. Implementation of Denoising Autoencoder

We built the DAE with PyTorch with reference to [40], and Table 4 and Table 5 show the network structure of the encoder and the decoder in the DAE, respectively. We utilized the structures shown in the tables because the implemented DAE with the encoder and decoder consisting of the three layers showed sufficient performance. The input data, including noise, is converted into the latent vector by passing through the layers of the encoder. The layers of the decoder are utilized to generate the new data using the latent vector as input. We implemented DAE where the original data with noise are input and training is performed in the direction of minimizing the error between the DAE’s output and the original data without noise. We set the hyperparameters as shown in Table 6 by referring to the values used for DAEs [40,41]. Like Table 3, Table 6 also does not show the size of the dataset and the number of epochs.

### 4.4. Implementation of Zero-Padding Technique

To eliminate the artifacts that occur in the generated data due to the Raman spectrum’s discontinuity at both ends, we implemented the system to add zero values of the determined length at both ends of the training data. After training using the zero-padded data, the system naturally generates the data longer than the original data. Thus, the system cuts both ends of the data by the padding length to remove the artifacts.

### 4.5. Implementation of the Proposed System including Federated Learning

We implemented the proposed system, including FL, referring to [42], to build the distributed data augmentation system. We built the system, including the aforementioned implementations on Ubuntu 20.04, using the desktop equipped with AMD Ryzen™ 7 5800X and 32 GB RAM. We trained the models in the system by utilizing NVIDIA’s compute unified device architecture (CUDA) on NVIDIA GeForce RTX 3070 8GB GDDR6 PCI Express 4.0 graphic card for faster learning.

## 5. Performance Evaluation

In this section, we first describe the various experiments for evaluating each technique that constitutes the proposed system and then show the evaluation results. Further, we explain the system’s performance from the perspective of diverse evaluation indices. In addition, we give explanations about the performance improvement when using FL and the analysis of the effectiveness of the proposed system. We conducted the above experiments and analyses using various chemicals, such as 2-chloroethyl ethyl sulfide (2-CEES), Dichlorvos (DDVP), Diisopropylfluorophosphate (DFP), and Dimethyl methyl phosphonate (DMMP). However, the experiments using different chemicals have the same conclusions, so this section includes only the results of experiments using 2-CEES.

### 5.1. Performance Evaluation of 1-D Signal GAN

**Figure 7:**
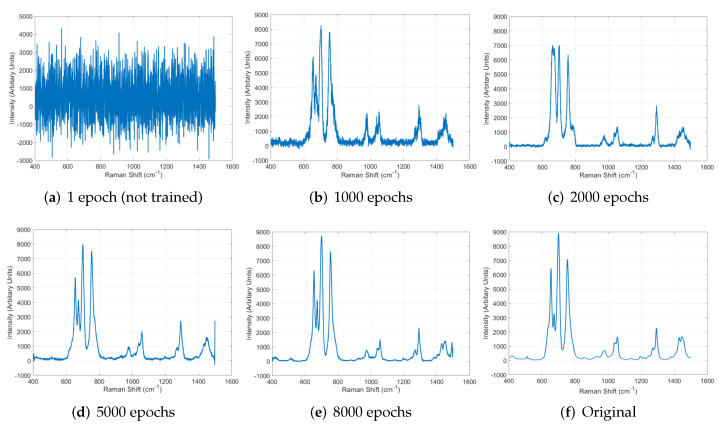
Raman spectrum data generated by GAN as the training progressed and original data.

We evaluated the performance of GAN described in Section 4.2. In this experiment, to clearly perform the evaluation, we conducted the training of GAN without including all the other techniques in the proposed system. Thus, the experiment was performed using only one real Raman spectrum data because the DA was not used in this experiment. While training GAN, we looked into the output data produced by the generator of GAN at 1 (not trained), 1000, 2000, 5000, and 8000 epochs. We plot the graphs using the output data, and Figure 7 shows the results and the original. As shown in the figure, the GAN generated data more similar to the original data as the training progressed, and the sufficiently trained GAN properly generated acceptable data mimicking the real data well. This result validates that the GAN in our system can be used as the fundamental technique to generate Raman spectrum data in large quantities from a small number of real data.

### 5.2. Evaluation of the Effect of Using Data Augmentation

**Figure 8:**
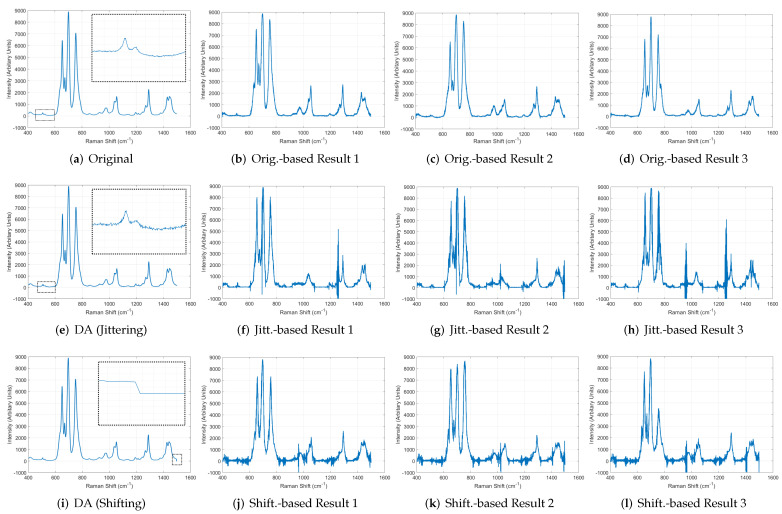
Performance comparison among GAN models trained using the original data and the augmented data with jittering or shifting.

As shown in Section 5.1, when the GAN was trained using a small number of real data, the GAN generated only data very similar to the original, which means there is low diversity in the GAN. Figure 8a–d in the first row show the original and the results generated by the GAN trained using the original, respectively. Such GAN is inappropriate to create data that maintains the characteristics of the original data but differs to some extent. Thus, as explained in Section 3.4, the system performs DA using small amounts of original data to secure sufficient amounts of initial data for training GAN, which improves the diversity of GAN. Figure 8e in the second row shows the data augmented using jittering, and Figure 8f–h present the results generated by the GAN trained using augmented data. In addition, as shown in Figure 8i, we conducted another augmentation by shifting the original data to the left or right randomly within the scale range of 10. The augmented data were used to train the GAN, and Figure 8j–l show the results generated by the trained GAN. As shown in the figures, the GAN generated much more diverse types of data when the GAN was trained using the augmented data than using only a small number of original data. In other words, the diversity of GAN improved by using the augmented data.

### 5.3. Performance Evaluation of Denoising Autoencoder

**Figure 9:**
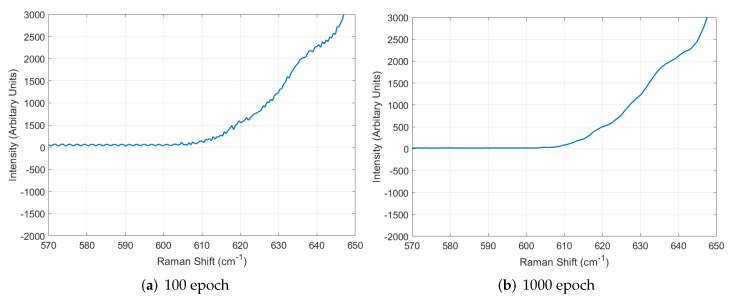
Performance analysis of DAE in terms of the amount of training.

**Figure 10:**
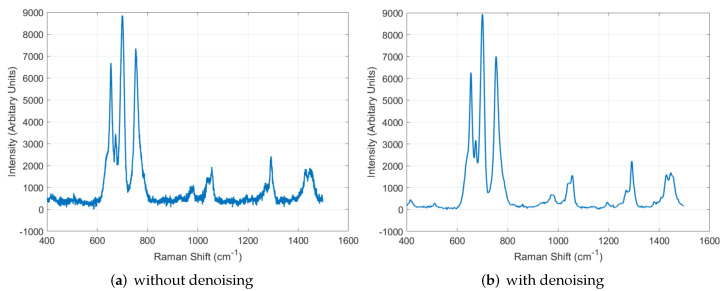
Performance comparison between the results with and without denoising.

The diversity of GAN was improved by using the augmented dataset, as explained in Section 5.2. However, the GAN generated data with amplified noise, so the proposed system utilized DAE to alleviate the exaggerated noise. To analyze the performance of DAE, we conducted the training using 100 data, including noise as input, and we looked into the results while training the DAE. Figure 9 shows the analysis of training, and we can see that the DAE removed noise better when training on 1000 epochs was performed. Thus, the proposed system utilized DAE that had performed training on 1000 epochs. Figure 10 shows the comparison between the results with and without using DAE, and we can see that the noise was alleviated well after denoising.

### 5.4. Evaluation of the Effect of Using the Zero-Padding Technique

**Figure 11:**
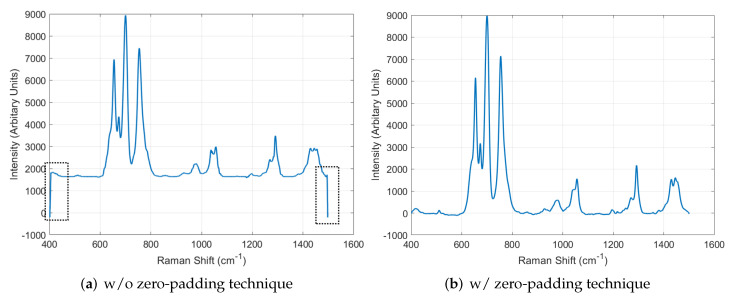
Evaluation of the effect of using the zero-padding technique.

Through the denoising process described in Section 5.3, the proposed system was able to generate a large number of data, including reasonable variations. However, at both ends of the generated data, there were artifacts that had not existed in the original data. Therefore, the system utilized the ZP technique to eliminate the artifacts in the last step described in Figure 4. Figure 11 shows the Raman spectrum data generated by the system with and without the ZP technique. As shown in Figure 11a, the values in the end regions soar when the ZP technique is not used. However, using the technique, the artifacts were eliminated well, so the system was able to obtain realistic Raman spectrum data.

### 5.5. Performance Analysis from the Perspective of Evaluation Indices

To further evaluate the performance of the proposed system, we analyzed the quality of Raman spectrum data generated by the system in terms of various evaluation indices. We utilized Fréchet inception distance (FID), Pearson correlation coefficient (PCC), and Euclidean distance (ED) as criteria to evaluate the quality of the generated data. FID is an index that measures the similarity of real data to generated data, and this index is frequently used to determine the quality of data created by GAN [43]. FID is the distance between the Gaussian with mean and covariance, (m,C), obtained from p(.) and the Gaussian $\left(m_{w},C_{w}\right)$ obtained from $p_{w}\left(.\right)$ as follows:

(10) $$d^{2}\left(\left(m,C\right),\left(m_{w},C_{w}\right)\right)=||m-m_{w}{\left|\right|}_{2}^{2}+\mathrm{Tr}\left(C+C_{w}-2{\left(CC_{w}\right)}^{1/2}\right).$$

p(.) and $p_{w}\left(.\right)$ denote the distribution of model samples and the distribution of the samples from the real world, respectively. A FID value closer to 0 means higher data quality. PCC is mainly used to evaluate the correlation between two variables, and the closer the correlation coefficient is to 1, the higher the similarity [44]. The PCC is calculated as follows:

(11) $$r=\frac{\sum \left(x-m_{x}\right)\left(y-m_{y}\right)}{\sqrt{\sum {\left(x-m_{x}\right)}^{2}\sum {\left(y-m_{y}\right)}^{2}}},$$

where $m_{x}$ is the mean of vector *x* and $m_{y}$ is the mean of the vector *y* [45]. ED is a method of obtaining the shortest distance between two points, and the closer the number is to zero, the smaller the distance between the two data is. The ED is calculated as follows:

(12) $$d\left(x,y\right)=\sqrt{\sum_{i=1}^{n}{\left(y_{i}-x_{i}\right)}^{2}}.$$

We implemented the source code for the measurement using NumPy [46] by referring to the work in [47].

Table 7 shows the result of the ablation study of the proposed system in terms of the aforementioned evaluation indices. As a result, the system using DA and DAE, applying ZP before GAN, and removing ZP in the last step showed the best performance. Thus, we designed the proposed system to perform the operations in such order.

### 5.6. Evaluation of Performance Improvement When Using Federated Learning

**Figure 12:**
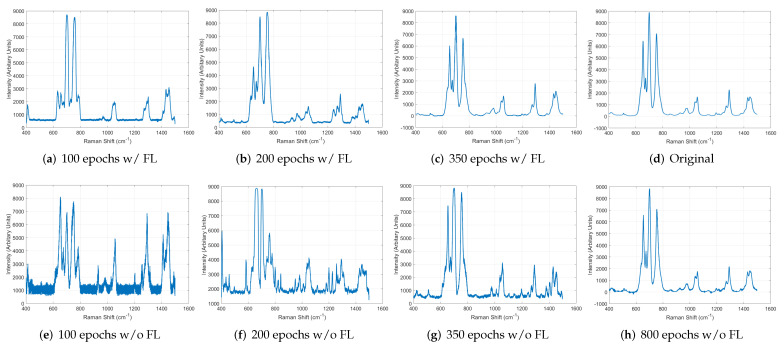
Performance comparison between the results with and without using FL.

The proposed system leverages FL to enable cooperation among decentralized troops and faster learning. To evaluate the performance improvement when using FL, we conducted the experiment, supposing that five troops performed FL. In this experiment, each troop had 100 augmented Raman spectrum data and conducted the training using FL on the proposed system. While training, we looked into the result data of one troop at 100, 200, and 350 epochs to analyze the training performance. Figure 12a–c show the results when the system utilized the FL. As shown in the figures, the training was performed much faster, and the result generated at the 350th epoch was sufficiently similar to the original presented in Figure 12d. On the contrary, without using the FL, the system performed training slowly, and the generated data after about 800 epochs were similar to the original. This experiment result proves that the proposed system can build the models more quickly and efficiently without exchanging raw data between the troops by leveraging FL, which improves the system’s practical feasibility on real battlefields.

### 5.7. Analysis of the Effectiveness of the Proposed System

**Figure 13:**
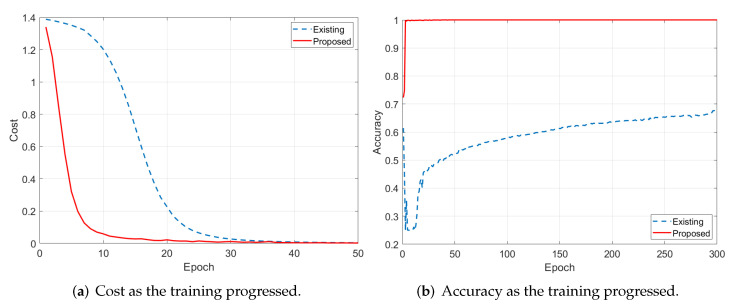
Performance analysis of the effectiveness of the proposed system.

Through Section 5.1, Section 5.2, Section 5.3, Section 5.4, Section 5.5 and Section 5.6, we validated that the proposed system trains the models quickly and generates realistic Raman spectrum data well. As we mentioned before, sufficient amounts of data for training are required to build the AI-based chemical agent detection system, so we devised the proposed system. Thus, in this subsection, we conducted an additional experiment to analyze the effect of the application of the proposed system. For this experiment, we built the convolutional neural network (CNN)-based model for chemical agent classification and trained the model using two datasets. One was the dataset based on the collected real data, and the other was composed of data generated by the proposed system. Naturally, the amounts of the real data were less than those of the generated data, which caused slow training. Thus, for fair evaluation, the real data were duplicated to equalize the amounts of data in the two datasets. Figure 13a shows the cost values in the training process, and we can see that the training was performed much faster when the proposed system was used. Further, as shown in Figure 13b, the classification model trained using the dataset generated by the proposed system outperformed the model that utilized the dataset based on the collected real data. Repeated use of only a small number of data caused a lack of diversity in training data, which resulted in the poor classification performance of the existing system. These results validated the effectiveness of the proposed system.

## 6. Conclusions

In this paper, we devised the distributed Raman spectrum data augmentation system. The proposed system utilizes not only deep generative models, such as GAN and DAE but also additional techniques, such as the random transformation-based DA and ZP, to generate Raman spectrum data properly. Furthermore, the system leverages FL to enable cooperation among decentralized troops and faster learning. We implemented the techniques that constitute our system and conducted various experiments to evaluate each technique. Further, we analyzed the performance of the proposed system from the perspective of diverse evaluation indices. Moreover, we proved the performance improvement by using FL and the effectiveness of the proposed system. Based on the evaluation results, we validated that the proposed system trains the models quickly and efficiently and generates realistic Raman spectrum data well.

We have several directions for future work. We plan to strengthen our system to generate Raman spectra of more diverse chemical agents by leveraging a conditional GAN. In addition, we will improve the system to be able to consider more various factors affecting Raman spectrum data, such as baseline drifts due to fluorescence or other reasons.

## Figures and Tables

**Table 1:** Network layer structure of discriminator in GAN used for the proposed system.

Layer	Composition	In Channel	Out Channel	Kernel Size	Stride	Padding	# Parameters
1	Conv1d	1	64	4	2	1	256
	LeakyReLU	-	-	-	-	-	-
2	Conv1d	64	128	4	2	1	32,768
	BatchNorm1d	-	-	-	-	-	256
	LeakyReLU	-	-	-	-	-	-
3	Conv1d	128	256	4	2	1	131,072
	BatchNorm1d	-	-	-	-	-	512
	LeakyReLU	-	-	-	-	-	-
4	Conv1d	256	512	4	2	1	524,288
	BatchNorm1d	-	-	-	-	-	1024
	LeakyReLU	-	-	-	-	-	-
5	Conv1d	512	1	datasize/16	1	0	86,016

**Table 2:** Network layer structure of the generator in GAN used for the proposed system.

Layer	Composition	In Channel	Out Channel	Kernel Size	Stride	Padding	# Parameters
1	ConvTranspose1d	noise size	512	datasize/16	1	0	8,601,600
	BatchNorm1d	-	-	-	-	-	1024
	ReLU	-	-	-	-	-	-
2	ConvTranspose1d	512	256	4	2	1	524,288
	BatchNorm1d	-	-	-	-	-	512
	LeakyReLU	-	-	-	-	-	-
3	ConvTranspose1d	256	128	4	2	1	131,072
	BatchNorm1d	-	-	-	-	-	256
	LeakyReLU	-	-	-	-	-	-
4	ConvTranspose1d	128	64	4	2	1	32,768
	BatchNorm1d	-	-	-	-	-	128
	LeakyReLU	-	-	-	-	-	-
5	ConvTranspose1d	64	1	4	2	1	256

**Table 3:** Hyperparameters and values used for GAN in the proposed system.

Hyperparameter	Value
Batch size	8
Learning rate	0.0001
Weight clipping	0.01
Discriminator updates per generate update	5
Optimizer algorithm	RMSprop

**Table 4:** Network layer structure of the encoder in DAE used for the proposed system.

Layer	Composition	In Channel	Out Channel	Kernel Size	Stride	# Parameters
1	Conv1d	1	4	4	1	20
2	Conv1d	4	16	4	1	272
3	Conv1d	16	32	4	1	2080

**Table 5:** Network layer structure of the decoder in DAE used for the proposed system.

Layer	Composition	In Channel	Out Channel	Kernel Size	# Parameters
1	ConvTranspose1d	32	16	4	2064
2	ConvTranspose1d	16	4	4	260
3	ConvTranspose1d	4	1	4	17

**Table 6:** Hyperparameters and values used for DAE in the proposed system.

Hyperparameter	Value
Batch size	10
Learning rate	0.001
Loss function	MSE
Optimizer algorithm	Adam

**Table 7:** Ablation study of the proposed system in terms of the evaluation indices.

System Composition	Evaluation Index		
	FID	PCC	ED
GAN	0.161	0.996	0.742
DA-GAN	0.324	0.992	1.025
DA-GAN-DAE	0.477	0.998	1.336
DA-ZP-GAN-RZP-DAE	0.070	0.999	0.508
DA-ZP-GAN-DAE-RZP	0.038	0.999	0.361

## Data Availability

Data sharing not applicable.

## Abbreviations

The following abbreviations are used in this manuscript:

AI	Artificial Intelligence
FL	Federated Learning
1-D	One-Dimensional
GAN	Generative Adversarial Network
DAE	Denoising Autoencoder
ZP	Zero Padding
DA	Data Augmentation
RNN	Recurrent Neural Network
LSTM	Long Short-Term Memory
GRU	Gated Recurrent Units
CUDA	Compute Unified Device Architecture
FID	Fréchet Inception Distance
PCC	Pearson Correlation Coefficient
ED	Euclidean Distance
CNN	Convolutional Neural Network
